# Dianilinium bis­(pyridine-2,6-dicarboxyl­ato-κ^3^ *O* ^2^,*N*,*O* ^6^)cuprate(II) hexa­hydrate

**DOI:** 10.1107/S1600536812028590

**Published:** 2012-07-04

**Authors:** Amir Shokooh Saljooghi, Hadi Amiri Rudbari, Francesco Nicolò, Maliheh Zahmati, Fatemeh Delavar Mendi, Hossein Eshtiagh-Hosseini, Masoud Mirzaei

**Affiliations:** a Department of Chemistry, Ferdowsi University of Mashhad, Mashhad 91779, Iran; b Dipartimento di Chimica Inorganica, Chimica Analitica e Chimica Fisica, Università di Messina, Salita Sperone, 31 Contrada Papardo, 98166 Messina, Italy

## Abstract

The asymmetric unit of the title complex, (C_6_H_8_N)_2_[Cu(C_7_H_3_NO_4_)_2_]·6H_2_O, contains half a copper(II)–dipicolinate complex located on a twofold rotation axis, one protonated aniline mol­ecule and three solvent water mol­ecules. The Cu^II^ atom is coordinated by four O atoms and two N atoms from two dipicolinate ligands in a distorted octa­hedral environment. In the crystal, the components are linked into a three-dimensional framework by inter­molecular O—H⋯O and N—H⋯O inter­actions.

## Related literature

For metal complexes formed by pyridine­dicarb­oxy­lic acids, see: Crans (2000 ▶); Wang *et al.* (2004 ▶); Park *et al.* (2007 ▶); Aghabozorg *et al.* (2008 ▶, 2011 ▶); Tabatabaee (2010 ▶).
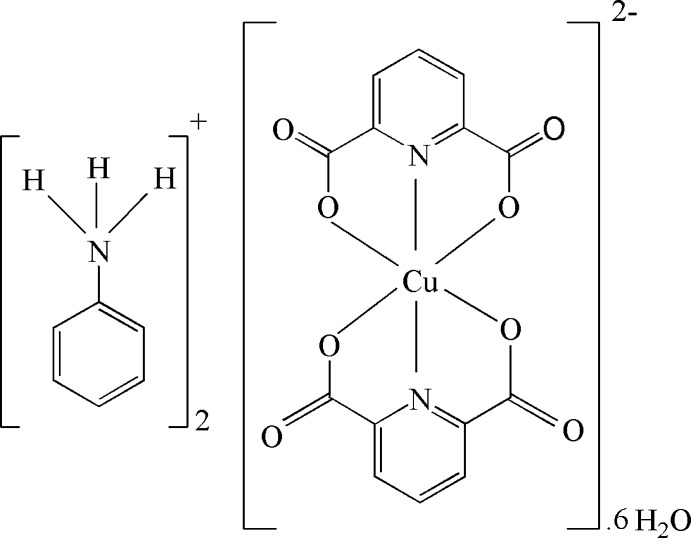


## Experimental

### 

#### Crystal data

(C_6_H_8_N)_2_[Cu(C_7_H_3_NO_4_)_2_]·6H_2_O
*M* *_r_* = 690.11
Monoclinic, 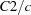

*a* = 20.9117 (6) Å
*b* = 7.9115 (2) Å
*c* = 19.8842 (5) Å
β = 117.706 (2)°
*V* = 2912.52 (13) Å^3^
*Z* = 4
Mo *K*α radiation
μ = 0.83 mm^−1^
*T* = 293 K
0.44 × 0.36 × 0.35 mm

#### Data collection

Bruker APEXII CCD diffractometer
Absorption correction: multi-scan (*SADABS*; Bruker, 2001 ▶) *T*_min_ = 0.652, *T*_max_ = 0.746
48649 measured reflections
5321 independent reflections
4990 reflections with *I* > 2σ(*I*)
*R*_int_ = 0.017

#### Refinement

*R*[*F* ^2^ > 2σ(*F* ^2^)] = 0.025
*wR*(*F* ^2^) = 0.073
*S* = 1.06
5321 reflections
213 parameters
H-atom parameters constrained
Δρ_max_ = 0.39 e Å^−3^
Δρ_min_ = −0.55 e Å^−3^

### 

Data collection: *APEX2* (Bruker, 2007 ▶); cell refinement: *SAINT* (Bruker, 2007 ▶); data reduction: *SAINT*; program(s) used to solve structure: *SHELXS97* (Sheldrick, 2008 ▶); program(s) used to refine structure: *SHELXL97* (Sheldrick, 2008 ▶); molecular graphics: *XPW* (Siemens, 1996 ▶); software used to prepare material for publication: *SHELXTL* (Sheldrick, 2008 ▶).

## Supplementary Material

Crystal structure: contains datablock(s) I, global. DOI: 10.1107/S1600536812028590/vn2037sup1.cif

Structure factors: contains datablock(s) I. DOI: 10.1107/S1600536812028590/vn2037Isup2.hkl

Additional supplementary materials:  crystallographic information; 3D view; checkCIF report

## Figures and Tables

**Table 1:** Selected bond lengths (Å)

Cu1—N1	1.9232 (7)
Cu1—O1	2.2577 (7)
Cu1—O2	2.1701 (7)

**Table 2:** Hydrogen-bond geometry (Å, °)

*D*—H⋯*A*	*D*—H	H⋯*A*	*D*⋯*A*	*D*—H⋯*A*
N2—H2*A*⋯O4^i^	0.83	1.91	2.7267 (11)	171
N2—H2*B*⋯O7^ii^	0.89	1.93	2.8071 (12)	168
N2—H2*C*⋯O3^iii^	0.88	2.14	2.9842 (12)	162
O5—H5*A*⋯O2^iv^	0.85	1.88	2.732	176
O5—H5*B*⋯O6^ii^	0.85	1.97	2.8026 (12)	166
O6—H6*A*⋯O3^v^	0.85	1.97	2.8066 (11)	167
O6—H6*B*⋯O5^vi^	0.85	1.93	2.7760 (13)	177
O7—H7*A*⋯O1^vii^	0.85	1.92	2.750	164
O7—H7*B*⋯O6^viii^	0.85	2.17	3.0083 (12)	170

Symmetry codes: (i) 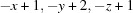; (ii) 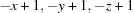; (iii) 
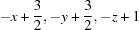
; (iv) 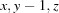; (v) 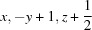; (vi) 
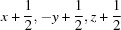
; (vii) 
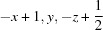
; (viii) 
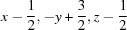
.

**Fig. 1.**
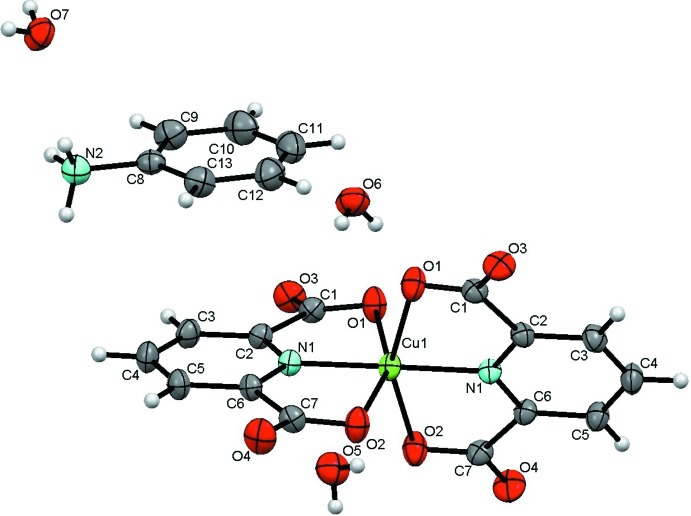
Molecular structure and atom labeling scheme for title compound with displacement ellipsoids at the 50% probability level.

**Fig. 2.**
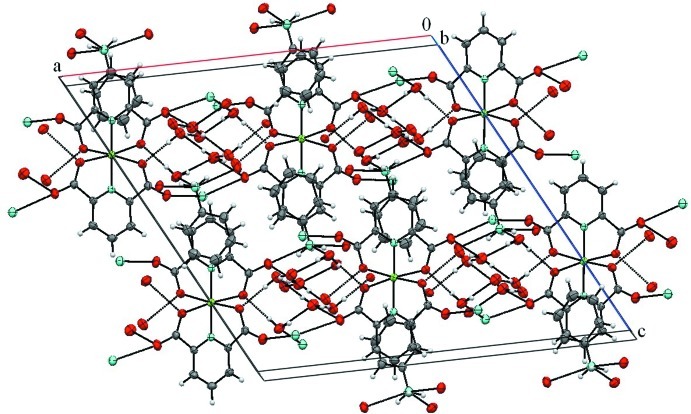
A view of H-bonded chain in crystal network of title compound, hydrogen bonds are shown as dashed lines.

## supplementary crystallographic information

### Comment

Pyridine-2,6-dicarboxylic acid (dipicolinic acid) is a versatile N—O donor capable of forming stable chelates (Park *et al.*, 2007), with various metal ions and it can exhibit diverse coordination modes such as monodentate (Park *et al.*, 2007), bidentate (Wang *et al.*, 2004), tridentate (Park *et al.*, 2007), meridian (Park *et al.*, 2007), or bridging (Aghabozorg *et al.*, 2008, 2011). Dipicolinic acid (H_2_dipic) and its anions (Hdipic^-^, dipic2^-^), have been extensively used in the design of coordination compounds, due to the variety of their bonding ability and the relatively strong hydrogen bonds they form. Dipicolinic acid is a beneficial compound for the human organism and it is involved in several essential biochemical processes. It shows various biological functions and is a suitable ligand for modeling potential pharmacological compounds because of its low toxicity and amphiphilic nature (Crans, 2000). In recent years, syntheses and crystal structures of a large number of complexes with dipicolinic acid and some amino compounds have been reported (Aghabozorg *et al.*, 2008; Tabatabaee, 2010). Here, we present the preparation and the crystal structure of the title compund, (C_6_H_8_N)_2_ [Cu(C_7_H_3_NO_4_)_2_].6H_2_O. The molecular structure of the title compound is shown in Fig. 1. The cationic portion of the asymmetric unit consists of one protonated aniline molecule (anilinium cation) and the anionic portion is the [Cu(pydc)_2_]^2-^ complex.In the anion, the angles O1—Cu1—O1 [103.65 (5)°], O2—Cu1—O2 [102.71 (5)°] and N1—Cu1—N1 [179.46 (5)°] indicate that the coordination environment around Cu(II) ion is a distorted octahedron. There are extensive intermolecular O—H···O, N—H···O and weak C—H···O hydrogen bonds, which increases the stability of the crystal structure (Fig. 2).

### Experimental

The title compound was synthesized by the reaction of copper(II) acetate, pyridine-2,6-dicarboxylic acid (pydcH_2_) and aniline in aqueous solution in a 1:1:1 molar ratio. Green crystals of the title compound were obtained by slow evaporation of the solvent at room temperature.

### Refinement

The H atoms of the water molecules were found in difference Fourier maps and the O–H bond lengths were constrained to 0.85 Å. The positions of the water molecules were optimized using rigid-body constraints (*SHELXL* AFIX 6). The H atoms from C–H groups were placed in calculated positions. The carbon H atoms were refined in riding model approximation with *U*_iso_(H) = 1.2*U*_eq_(C) whereas the water hydrogens were treated with 1.5*U*_eq_(O).

### Crystal data

**Table:**

(C_6_H_8_N)_2_[Cu(C_7_H_3_NO_4_)_2_]·6H_2_O	*F*(000) = 1436
*M**_r_* = 690.11	*D*_x_ = 1.574 Mg m^−^^3^
Monoclinic, *C*2/*c*	Mo *K*α radiation, λ = 0.71073 Å
Hall symbol: -C 2yc	Cell parameters from 9787 reflections
*a* = 20.9117 (6) Å	θ = 2.3–32.7°
*b* = 7.9115 (2) Å	µ = 0.83 mm^−^^1^
*c* = 19.8842 (5) Å	*T* = 293 K
β = 117.706 (2)°	Irregular, green
*V* = 2912.52 (13) Å^3^	0.44 × 0.36 × 0.35 mm
*Z* = 4	

### Data collection

**Table:**

Bruker APEXII CCD diffractometer	5321 independent reflections
Radiation source: fine-focus sealed tube	4990 reflections with *I* > 2σ(*I*)
Graphite monochromator	*R*_int_ = 0.017
φ and ω scans	θ_max_ = 32.7°, θ_min_ = 2.2°
Absorption correction: multi-scan (*SADABS*; Bruker, 2001)	*h* = −31→31
*T*_min_ = 0.652, *T*_max_ = 0.746	*k* = −12→12
48649 measured reflections	*l* = −30→30

### Refinement

**Table:**

Refinement on *F*^2^	Primary atom site location: structure-invariant direct methods
Least-squares matrix: full	Secondary atom site location: difference Fourier map
*R*[*F*^2^ > 2σ(*F*^2^)] = 0.025	Hydrogen site location: inferred from neighbouring sites
*wR*(*F*^2^) = 0.073	H-atom parameters constrained
*S* = 1.06	*w* = 1/[σ^2^(*F*_o_^2^) + (0.0415*P*)^2^ + 1.1609*P*] where *P* = (*F*_o_^2^ + 2*F*_c_^2^)/3
5321 reflections	(Δ/σ)_max_ = 0.001
213 parameters	Δρ_max_ = 0.39 e Å^−^^3^
0 restraints	Δρ_min_ = −0.55 e Å^−^^3^

### Special details

**Table:**

Geometry. All esds (except the esd in the dihedral angle between two l.s. planes) are estimated using the full covariance matrix. The cell esds are taken into account individually in the estimation of esds in distances, angles and torsion angles; correlations between esds in cell parameters are only used when they are defined by crystal symmetry. An approximate (isotropic) treatment of cell esds is used for estimating esds involving l.s. planes.
Refinement. Refinement of *F*^2^ against ALL reflections. The weighted *R*-factor *wR* and goodness of fit *S* are based on *F*^2^, conventional *R*-factors *R* are based on *F*, with *F* set to zero for negative *F*^2^. The threshold expression of *F*^2^ > 2σ(*F*^2^) is used only for calculating *R*-factors(gt) *etc*. and is not relevant to the choice of reflections for refinement. *R*-factors based on *F*^2^ are statistically about twice as large as those based on *F*, and *R*- factors based on ALL data will be even larger.

### Fractional atomic coordinates and isotropic or equivalent isotropic displacement parameters (Å^2^)

**Table:**

	*x*	*y*	*z*	*U*_iso_*/*U*_eq_	
Cu1	0.5000	0.979382 (19)	0.2500	0.02257 (5)	
O1	0.59158 (4)	0.80292 (10)	0.26847 (4)	0.03298 (14)	
O2	0.43941 (4)	1.15066 (10)	0.28429 (4)	0.03275 (14)	
O3	0.70644 (4)	0.77108 (10)	0.35839 (4)	0.03315 (14)	
O4	0.43162 (4)	1.20999 (11)	0.39036 (4)	0.03787 (16)	
N1	0.55834 (4)	0.98045 (9)	0.35855 (4)	0.02101 (12)	
C1	0.64268 (4)	0.81793 (11)	0.33490 (5)	0.02413 (14)	
C2	0.62266 (4)	0.90375 (10)	0.39060 (4)	0.02198 (13)	
C3	0.66492 (5)	0.90453 (12)	0.46879 (5)	0.02786 (15)	
H3	0.7102	0.8535	0.4911	0.033*	
C4	0.63852 (5)	0.98284 (13)	0.51321 (5)	0.03126 (18)	
H4	0.6656	0.9822	0.5658	0.038*	
C5	0.57176 (5)	1.06210 (12)	0.47912 (5)	0.02811 (16)	
H5	0.5534	1.1150	0.5082	0.034*	
C6	0.53317 (4)	1.06049 (11)	0.40074 (4)	0.02196 (13)	
C7	0.46167 (4)	1.14859 (11)	0.35519 (5)	0.02438 (14)	
N2	0.65628 (5)	0.54403 (11)	0.60233 (5)	0.03183 (16)	
H2A	0.6328	0.6190	0.6098	0.038*	
H2B	0.6526	0.4490	0.6238	0.038*	
H2C	0.7012	0.5780	0.6201	0.038*	
C8	0.63075 (5)	0.51163 (11)	0.52136 (6)	0.02671 (15)	
C9	0.67563 (6)	0.42455 (14)	0.50001 (6)	0.03540 (19)	
H9	0.7207	0.3871	0.5364	0.042*	
C10	0.65261 (7)	0.39375 (16)	0.42367 (7)	0.0440 (2)	
H10	0.6826	0.3359	0.4087	0.053*	
C11	0.58577 (8)	0.44806 (16)	0.36981 (7)	0.0442 (3)	
H11	0.5706	0.4266	0.3186	0.053*	
C12	0.54110 (7)	0.53474 (16)	0.39200 (7)	0.0418 (2)	
H12	0.4959	0.5711	0.3556	0.050*	
C13	0.56340 (5)	0.56767 (14)	0.46834 (6)	0.03397 (18)	
H13	0.5337	0.6262	0.4834	0.041*	
O5	0.29295 (4)	0.17557 (11)	0.19901 (5)	0.03988 (17)	
H5A	0.3387	0.1722	0.2247	0.060*	
H5B	0.2852	0.2755	0.2092	0.060*	
O6	0.74953 (5)	0.49186 (10)	0.79362 (5)	0.04002 (17)	
H6A	0.7296	0.4169	0.8082	0.060*	
H6B	0.7635	0.4372	0.7660	0.060*	
O7	0.37393 (5)	0.76400 (11)	0.34828 (4)	0.03909 (16)	
H7A	0.3829	0.7562	0.3108	0.059*	
H7B	0.3414	0.8380	0.3387	0.059*	

### Atomic displacement parameters (Å^2^)

**Table:**

	*U*^11^	*U*^22^	*U*^33^	*U*^12^	*U*^13^	*U*^23^
Cu1	0.01931 (7)	0.02986 (8)	0.01981 (7)	0.000	0.01017 (5)	0.000
O1	0.0284 (3)	0.0478 (4)	0.0232 (3)	0.0025 (3)	0.0124 (2)	−0.0029 (3)
O2	0.0236 (3)	0.0484 (4)	0.0236 (3)	0.0075 (3)	0.0088 (2)	0.0010 (3)
O3	0.0245 (3)	0.0373 (4)	0.0392 (3)	0.0072 (3)	0.0161 (3)	−0.0025 (3)
O4	0.0355 (3)	0.0459 (4)	0.0394 (4)	0.0126 (3)	0.0236 (3)	−0.0026 (3)
N1	0.0190 (3)	0.0251 (3)	0.0198 (3)	0.0023 (2)	0.0097 (2)	0.0004 (2)
C1	0.0232 (3)	0.0254 (3)	0.0259 (3)	0.0028 (3)	0.0133 (3)	0.0011 (3)
C2	0.0193 (3)	0.0245 (3)	0.0222 (3)	0.0025 (2)	0.0097 (2)	0.0014 (2)
C3	0.0229 (3)	0.0320 (4)	0.0237 (3)	0.0049 (3)	0.0068 (3)	0.0031 (3)
C4	0.0306 (4)	0.0393 (5)	0.0188 (3)	0.0029 (3)	0.0071 (3)	0.0008 (3)
C5	0.0293 (4)	0.0348 (4)	0.0213 (3)	0.0014 (3)	0.0127 (3)	−0.0027 (3)
C6	0.0204 (3)	0.0261 (3)	0.0209 (3)	0.0009 (3)	0.0109 (3)	−0.0012 (3)
C7	0.0212 (3)	0.0271 (4)	0.0262 (3)	0.0017 (3)	0.0122 (3)	−0.0021 (3)
N2	0.0293 (4)	0.0354 (4)	0.0315 (4)	0.0046 (3)	0.0147 (3)	−0.0007 (3)
C8	0.0267 (4)	0.0248 (3)	0.0313 (4)	−0.0010 (3)	0.0157 (3)	0.0005 (3)
C9	0.0346 (5)	0.0356 (5)	0.0415 (5)	0.0057 (4)	0.0223 (4)	0.0012 (4)
C10	0.0554 (7)	0.0432 (6)	0.0474 (6)	0.0012 (5)	0.0357 (5)	−0.0046 (5)
C11	0.0581 (7)	0.0425 (6)	0.0352 (5)	−0.0113 (5)	0.0244 (5)	−0.0043 (4)
C12	0.0367 (5)	0.0438 (6)	0.0364 (5)	−0.0043 (4)	0.0098 (4)	0.0018 (4)
C13	0.0272 (4)	0.0349 (4)	0.0386 (5)	0.0013 (3)	0.0144 (4)	−0.0004 (4)
O5	0.0276 (3)	0.0430 (4)	0.0443 (4)	0.0022 (3)	0.0128 (3)	0.0012 (3)
O6	0.0493 (5)	0.0317 (4)	0.0429 (4)	−0.0055 (3)	0.0246 (4)	−0.0021 (3)
O7	0.0470 (4)	0.0429 (4)	0.0332 (3)	0.0045 (3)	0.0235 (3)	0.0054 (3)

### Geometric parameters (Å, º)

**Table:**

Cu1—N1	1.9232 (7)	N2—C8	1.4641 (13)
Cu1—N1^i^	1.9232 (7)	N2—H2A	0.8262
Cu1—O2^i^	2.1701 (7)	N2—H2B	0.8855
Cu1—O1	2.2577 (7)	N2—H2C	0.8779
Cu1—O2	2.1701 (7)	C8—C9	1.3807 (13)
Cu1—O1^i^	2.2577 (7)	C8—C13	1.3823 (14)
O1—C1	1.2603 (10)	C9—C10	1.3841 (16)
O2—C7	1.2628 (10)	C9—H9	0.9300
O3—C1	1.2467 (10)	C10—C11	1.376 (2)
O4—C7	1.2364 (10)	C10—H10	0.9300
N1—C2	1.3365 (10)	C11—C12	1.3859 (19)
N1—C6	1.3389 (10)	C11—H11	0.9300
C1—C2	1.5146 (11)	C12—C13	1.3897 (16)
C2—C3	1.3858 (11)	C12—H12	0.9300
C3—C4	1.3865 (13)	C13—H13	0.9300
C3—H3	0.9300	O5—H5A	0.8500
C4—C5	1.3863 (13)	O5—H5B	0.8500
C4—H4	0.9300	O6—H6A	0.8502
C5—C6	1.3823 (11)	O6—H6B	0.8500
C5—H5	0.9300	O7—H7A	0.8499
C6—C7	1.5109 (11)	O7—H7B	0.8500
			
N1—Cu1—N1^i^	179.49 (4)	C4—C5—H5	120.8
N1—Cu1—O2^i^	101.04 (3)	N1—C6—C5	121.15 (8)
N1^i^—Cu1—O2^i^	78.64 (3)	N1—C6—C7	114.23 (7)
N1—Cu1—O2	78.64 (3)	C5—C6—C7	124.61 (7)
N1^i^—Cu1—O2	101.04 (3)	O4—C7—O2	127.31 (8)
O2^i^—Cu1—O2	102.72 (4)	O4—C7—C6	117.63 (8)
N1—Cu1—O1^i^	103.46 (3)	O2—C7—C6	115.06 (7)
N1^i^—Cu1—O1^i^	76.86 (3)	C8—N2—H2A	112.3
O2^i^—Cu1—O1^i^	155.50 (2)	C8—N2—H2B	108.2
O2—Cu1—O1^i^	82.08 (3)	H2A—N2—H2B	109.3
N1—Cu1—O1	76.86 (3)	C8—N2—H2C	105.7
N1^i^—Cu1—O1	103.46 (3)	H2A—N2—H2C	108.8
O2^i^—Cu1—O1	82.08 (3)	H2B—N2—H2C	112.5
O2—Cu1—O1	155.50 (2)	C9—C8—C13	121.51 (10)
O1^i^—Cu1—O1	103.61 (4)	C9—C8—N2	118.23 (9)
C1—O1—Cu1	110.73 (6)	C13—C8—N2	120.26 (8)
C7—O2—Cu1	112.14 (5)	C8—C9—C10	119.05 (10)
C2—N1—C6	121.17 (7)	C8—C9—H9	120.5
C2—N1—Cu1	120.39 (5)	C10—C9—H9	120.5
C6—N1—Cu1	118.44 (5)	C11—C10—C9	120.53 (11)
O3—C1—O1	127.06 (8)	C11—C10—H10	119.7
O3—C1—C2	118.06 (7)	C9—C10—H10	119.7
O1—C1—C2	114.87 (7)	C10—C11—C12	119.86 (11)
N1—C2—C3	120.56 (7)	C10—C11—H11	120.1
N1—C2—C1	114.45 (7)	C12—C11—H11	120.1
C3—C2—C1	124.99 (7)	C11—C12—C13	120.45 (11)
C2—C3—C4	118.79 (8)	C11—C12—H12	119.8
C2—C3—H3	120.6	C13—C12—H12	119.8
C4—C3—H3	120.6	C8—C13—C12	118.59 (10)
C5—C4—C3	119.95 (8)	C8—C13—H13	120.7
C5—C4—H4	120.0	C12—C13—H13	120.7
C3—C4—H4	120.0	H5A—O5—H5B	99.9
C6—C5—C4	118.33 (8)	H6A—O6—H6B	103.6
C6—C5—H5	120.8	H7A—O7—H7B	109.5
			
N1—Cu1—O1—C1	−14.29 (6)	O3—C1—C2—C3	−13.90 (13)
N1^i^—Cu1—O1—C1	165.31 (6)	O1—C1—C2—C3	166.50 (9)
O2^i^—Cu1—O1—C1	89.06 (6)	N1—C2—C3—C4	1.50 (13)
O2—Cu1—O1—C1	−14.29 (11)	C1—C2—C3—C4	−177.53 (9)
O1^i^—Cu1—O1—C1	−115.21 (7)	C2—C3—C4—C5	−1.60 (15)
N1—Cu1—O2—C7	−11.16 (6)	C3—C4—C5—C6	−0.05 (15)
N1^i^—Cu1—O2—C7	169.24 (6)	C2—N1—C6—C5	−2.07 (13)
O2^i^—Cu1—O2—C7	−110.06 (7)	Cu1—N1—C6—C5	178.37 (7)
O1^i^—Cu1—O2—C7	94.37 (6)	C2—N1—C6—C7	176.68 (7)
O1—Cu1—O2—C7	−11.16 (11)	Cu1—N1—C6—C7	−2.87 (9)
O2^i^—Cu1—N1—C2	−71.51 (7)	C4—C5—C6—N1	1.91 (14)
O2—Cu1—N1—C2	−172.44 (7)	C4—C5—C6—C7	−176.72 (9)
O1^i^—Cu1—N1—C2	108.66 (6)	Cu1—O2—C7—O4	−167.46 (8)
O1—Cu1—N1—C2	7.56 (6)	Cu1—O2—C7—C6	12.58 (9)
O2^i^—Cu1—N1—C6	108.06 (6)	N1—C6—C7—O4	172.64 (8)
O2—Cu1—N1—C6	7.12 (6)	C5—C6—C7—O4	−8.65 (13)
O1^i^—Cu1—N1—C6	−71.77 (7)	N1—C6—C7—O2	−7.39 (11)
O1—Cu1—N1—C6	−172.88 (7)	C5—C6—C7—O2	171.32 (9)
Cu1—O1—C1—O3	−162.04 (8)	C13—C8—C9—C10	0.37 (16)
Cu1—O1—C1—C2	17.52 (9)	N2—C8—C9—C10	−179.56 (10)
C6—N1—C2—C3	0.33 (12)	C8—C9—C10—C11	−0.45 (18)
Cu1—N1—C2—C3	179.88 (6)	C9—C10—C11—C12	0.19 (19)
C6—N1—C2—C1	179.46 (7)	C10—C11—C12—C13	0.16 (18)
Cu1—N1—C2—C1	−0.99 (10)	C9—C8—C13—C12	−0.03 (15)
O3—C1—C2—N1	167.02 (8)	N2—C8—C13—C12	179.90 (9)
O1—C1—C2—N1	−12.58 (11)	C11—C12—C13—C8	−0.24 (17)

Symmetry code: (i) −*x*+1, *y*, −*z*+1/2.

### Hydrogen-bond geometry (Å, º)

**Table:**

*D*—H···*A*	*D*—H	H···*A*	*D*···*A*	*D*—H···*A*
N2—H2*A*···O4^ii^	0.83	1.91	2.7267 (11)	171
N2—H2*B*···O7^iii^	0.89	1.93	2.8071 (12)	168
N2—H2*C*···O3^iv^	0.88	2.14	2.9842 (12)	162
O5—H5*A*···O2^v^	0.85	1.88	2.732	176
O5—H5*B*···O6^iii^	0.85	1.97	2.8026 (12)	166
O6—H6*A*···O3^vi^	0.85	1.97	2.8066 (11)	167
O6—H6*B*···O5^vii^	0.85	1.93	2.7760 (13)	177
O7—H7*A*···O1^i^	0.85	1.92	2.750	164
O7—H7*B*···O6^viii^	0.85	2.17	3.0083 (12)	170

Symmetry codes: (i) −*x*+1, *y*, −*z*+1/2; (ii) −*x*+1, −*y*+2, −*z*+1; (iii) −*x*+1, −*y*+1, −*z*+1; (iv) −*x*+3/2, −*y*+3/2, −*z*+1; (v) *x*, *y*−1, *z*; (vi) *x*, −*y*+1, *z*+1/2; (vii) *x*+1/2, −*y*+1/2, *z*+1/2; (viii) *x*−1/2, −*y*+3/2, *z*−1/2.
